# Climate and sino-nasal symptoms

**DOI:** 10.1007/s00484-026-03154-z

**Published:** 2026-05-19

**Authors:** Sebastian S. Dobrow, Robert E. Davis, Katherine E. Franklin, Julian De La Chapa, Andrew Strumpf, Spencer C. Payne, Jose L. Mattos

**Affiliations:** 1 University of Virginia School of Medicine, 200 Jeanette Lancaster Way, Charlottesville, VA 22903 USA; 2 Department of General Surgery, Rush University Medical Center, 1620 W. Harrison St, Chicago, IL 60612 USA; 3 Department of Internal Medicine, University of Chicago Medicine, 5841 S. Maryland Ave., Chicago, 60637 IL USA; 4 Department of Environmental Sciences, University of Virginia, P.O. Box 400123, Charlottesville, VA 22904-4123 USA; 5 Department of Otolaryngology – Head and Neck Surgery, University of Virginia, P.O. Box 800713, Charlottesville, VA 22908 USA; 6 Division of Rhinology and Sinus Surgery, Department of Otolaryngology – Head and Neck Surgery, University of Virginia, P.O. Box 800713, Charlottesville, VA 22908 USA; 7 Department of Surgery & Perioperative Care, University of Texas at Austin Dell Medical School, Austin, TX 78172 USA

**Keywords:** SNOT-22, Spatial synoptic climatology, Sino-nasal symptoms, Rhinology, Sinusitis

## Abstract

Patients with sino-nasal issues often believe their symptoms are exacerbated by certain weather conditions, but these relationships are largely anecdotal. Using an extensive archive of patient surveys at the University of Virginia Rhinology Clinic from January 2010–March 2020, we examined whether sino-nasal symptoms were impacted by the weather on, or shortly before, the day of clinic visit. Clinic patients completed the Sino-Nasal Outcome Test (SNOT-22), a 22-question survey related to symptoms and severity, on the day of each visit. Included patients lived in one of 28 zip codes comprising the city of Charlottesville and Albemarle County. The primary exposure variable was the Spatial Synoptic Classification (SSC)—a nominal classification of each day’s weather type. The SSC weather type on the day of the clinic visit and up to three days before was used as the exposure variable. Weather on days with poor symptoms (mean daily SNOT-22 score ≥ 50) were compared to all other days via odds ratios (ORs) for lags of 0–3 days, and a multivariate mixed effects logistic regression was employed to examine the association between SSC and symptom severity. A total of 955 SNOT-22 surveys were acquired from 481 patients. Dry, polar days (SSC category “DP”) and days with transitional weather (SSC category “TR”) had 2.0 times the odds of high SNOT-22 scores compared to all other weather types on the day of clinic visit (OR 2.0 [95% CIs 1.4, 2.8]). Logistic regression results indicate 4.0 times higher odds of high SNOT-22 scores on DP days compared to dry moderate days. Climate’s effect on sinus symptoms should be considered when managing and measuring sino-nasal symptoms or treatment outcomes.

**Supplementary Information:**

The online version contains supplementary material available at 10.1007/s00484-026-03154-z.

## Introduction and background

Patients often attribute sino-nasal symptoms to changes in the weather (Gryglas 2016; Levine et al., 2014; Rudmik et al. 2015). In one study illustrating this perceived association, the Sinus, Allergy and Migraine Study enrolled patients with self-diagnosed ‘sinus headache’ and formally diagnosed them according to International Headache Society criteria (Eross et al. 2007). Of the included patients, 83% believed they had sinus headaches because they noticed that changes in weather impacted their symptoms. Previous investigation has demonstrated associations between environmental allergens, pollutants and sino-nasal symptoms (Mady et al. 2018a, b; Velasquez et al. 2020), as well as biochemical inflammatory markers (Cho et al. 2014; Kopp et al. 1999; Xian et al., 2020; Zhao et al. 2011).

The impact of climate on sino-nasal symptomatology has received little scientific attention, however. Environmental chamber experiments with a group of non-allergic rhinitis subjects observed adverse responses to both cold, dry air and to short-term (30- and 60-minute) temperature changes (Bernstein et al. 2012). Graudenz et al. (2006) measured the effect of exposure to cold, air-conditioned air on symptoms and inflammatory mediators in both allergic rhinitis patients and healthy controls. They found both symptoms and inflammation to be exacerbated by exposure to cold in both groups, with larger effect size in the allergic rhinitis group. del Rio et al. (2012) examined the effect of season on incidental sinus abnormality on magnetic resonance imaging (MRI, as measured by a Lund Mackay score > 0) in adults examined for non-sinus indications. They found more sinus abnormalities in winter versus summer in all patients and those with symptomatic sinus disease, and they concluded that winter’s colder, dryer conditions and higher levels of environmental pollutants (NO_2_, CO, air particle index) likely contributed to the observed increase in sinus pathology. Finally, in a multi-country study of patients with “empty nose syndrome” (a post-surgical sense of nasal obstruction despite demonstrably clear airway passages), Manji et al. (2019) found that study participants reported symptom exacerbations associated with dry air, air conditioning, and transitions associated with seasonal changes and moving between indoor and outdoor environments.

To explore possible associations between climate and sino-nasal symptoms, we merged rhinology patient survey and weather data over a 10-year period in Charlottesville, Virginia. Sino-nasal symptoms were measured by the Sino-Nasal Outcome Test (SNOT-22), a robust, validated, and commonly used 22-question survey that measures symptom severity on a 6-point Likert-scale across 5 subdomains: rhinologic, extranasal rhinologic (i.e., cough and nasal discharge), otologic/facial pain, emotional symptoms, and sleep (DeConde et al. 2014; Hopkins et al. 2009). The goal of this research is to identify relationships between patient self-reported sino-nasal symptoms and climatic factors at or before the time of clinic visit.

## Materials and methods

### Clinical patient data

Patients who saw any of four University of Virginia Medical Center rhinology providers between the dates January 1, 2010, and March 31, 2020, were included in the initial study population. To ensure that the weather experienced by included patients was similar, a geo-parameter was applied to include only patients with home addresses in one of the 28 zip codes (covering 736 square miles) comprising the city of Charlottesville, where the clinic is located, and surrounding Albemarle County, Virginia.

Upon each visit, clinic patients were asked to complete the Sino-Nasal Outcome Test (SNOT-22), a 22-question survey of symptom severity using a 6-point scale (from 0 “No problem” to 5 “Problem is as bad as it can be”) (Supplemental Table 1). The SNOT-22 is a commonly used and validated survey instrument to assess the impact of sino-nasal symptoms on a patient’s quality of life (Hopkins et al. 2009). The 22 questions can be grouped into 5 subdomains: rhinological, extranasal, ear/facial, psychological, and sleep. Higher scores indicate greater symptom severity and impact on quality of life, with a maximum score of 110.

Patients meeting these parameters were then stratified into International Classification of Diseases Versions 9 or 10 (ICD-9/10) based diagnosis groups as available in the electronic medical record. These groups included patients ever diagnosed with acute sinusitis, chronic rhinitis, allergic or non-allergic chronic rhinitis, chronic rhinosinusitis, chronic rhinosinusitis with nasal polyposis (CRSwNP) or without nasal polyposis (CRSsNP), structural issues (e.g. deviated septum), or “none of the above.”

### Weather data

Meteorological observations were sampled daily at 1 a.m., 7 a.m., 1 p.m., and 7 p.m. Eastern Standard Time from the weather station located at the Charlottesville-Albemarle County Airport from online sources. These observations are part of the Automated Surface Observing Systems (ASOS), a joint program run by the National Weather Service, Federal Aviation Administration, and Department of Defense that provides data on a variety of weather-related variables. Retrieved variables included air temperature, wet bulb temperature, dew point temperature, sea-level pressure, relative humidity, wind speed, and wind direction. From these, several derived variables were computed to account for the joint effects of temperature and humidity: apparent temperature, humidex, and the temperature-humidity index (Davis et al. 2016). In addition, the Wind Chill Index was calculated according to Osczevski and Bluestein (2005). Linear interpolation from adjacent measurements was used for single missing observations; two or more consecutive observations were coded as missing.

Additionally, hourly average levels of environmental ozone (O_3_) and particulate matter 2.5 microns in diameter or smaller (PM_2.5_) were measured at a monitoring site within the included geo-footprint by the Virginia Department of Environmental Quality. From these hourly data, 24-hour and daytime (7 a.m–6 p.m.) mean, maximum, and minimum values of each pollutant were calculated. Missing air quality data were not filled or interpolated.

### Preliminary data analysis

The survey data are limited to clinic days and are thus temporally inconsistent, with no data on weekends, holidays or other off days. Furthermore, daily sample sizes (i.e., the number of patients per day) tend to be small. These factors made it difficult to use time series regression-based approaches, such as generalized additive or distributed lag models, for data analysis. Instead, a scoping study was first conducted to determine general relationships between individual environmental variables and mean daily SNOT-22 scores. These results then motivated our ultimate analysis, as explained below.

The continuous SNOT-22 score variable was dichotomized by symptom severity using a previously validated threshold of 50 (the 83.6 percentile for our data), such that a SNOT-22 score ≥ 50 represents a “high” score and < 50 represents a “low” score (Toma and Hopkins 2016; Mady et al. 2018b). Independent, two-tailed t-tests were calculated for each weather variable and binary SNOT-22 score, while one-tailed t-tests were used for pollutant levels (with the hypothesis that poor air quality would exacerbate symptoms). Analyses were completed at lags of 0–3 days. A lagged association is one that relates an outcome with a prior exposure. For example, associating SNOT-22 scores with same-day weather represents a 0-day lag, while associating SNOT-22 results with weather two days prior to survey completion represents a 2-day lag.

Preliminary t-test results revealed a statistically significant association between high SNOT-22 days and various weather variables including low values of temperature, wet bulb temperature, dew point temperature, apparent temperature, humidex, and high values of sea-level pressure (Supplemental Table 2). Given the inherent climatological (and statistical) association between many of these variables, further analyses were conducted using a synoptic climatological approach.

### Spatial synoptic classification

Rather than studying weather by occurrence of individual weather variables (i.e., temperature, humidity, wind, pressure, etc.), a synoptic climatological approach considers the frequently recurring combinations of these factors as they are interrelated. This approach is comparable to examining weather variations in air masses, or large bodies of air that exhibit similar temperature and humidity characteristics over large geographic regions. The Spatial Synoptic Classification (SSC), used previously in numerous weather-related health studies (e.g., Sheridan and Kalkstein 2010; Davis et al. 2012; Lee et al. 2012; Hondula et al. 2013; Cakmak and Hebbern 2014), includes six distinct nominal weather types and one transitional category (Sheridan 2002). These weather types are dry polar (DP), dry moderate (DM), dry tropical (DT), moist polar (MP), moist moderate (MM), moist tropical (MT), and transitional (TR). Brief descriptions of these SSC categories can be found in Table 1.

**Table 1:** Spatial synoptic classification (SSC) categories and descriptions

SSC Weather Type	Description
Dry, polar (DP)	The coldest air mass, often associated with polar high-pressure systems and clear skies.
Dry, moderate (DM)	Mild and dry air, often linked to more extreme air masses that have modified over time and space.
Dry, tropical (DT)	Very hot and dry, desert-like air mass.
Moist, polar (MP)	Cloudy, humid, and cool conditions often associated with precipitation.
Moist, moderate (MM)	More humid and much warmer than MP, often occurring on MP’s equatorward flank.
Moist, tropical (MT)	Warmest and most humid air mass that is linked to oppressive weather and convective precipitation, especially in summer.
Transitional (TR)	Days in which one of the above weather types yields to another, classified based on the change in temperature, humidity, and pressure over a 24-hour period. These are typically days with frontal passages, often bringing changes of temperature, high wind speeds, and precipitation.

The SSC is a relative, seasonally adjusted classification in which each weather type can occur throughout the year. For example, summer DP days will be warmer and more humid than winter DP weather at a given location (Sheridan 2002). A calendar assigning one SSC type per day for the Charlottesville weather station is accessible from a website maintained by the SSC’s author (Sheridan 2024; http://sheridan.geog.kent.edu/ssc.html).

### Final statistical methods

One-way analysis of variance (ANOVA) was used to compare daily mean SNOT-22 scores as a function of day of week, month, year, and SSC group. T-tests were performed comparing means of numerical weather and pollutant variables on high and low SNOT-22 days. Chi-square tests were used to examine SSC frequencies associated with high vs. low SNOT-22 days, and odds ratios were computed from the associated 2 × 2 contingency tables. We then employed a logistic regression model with multivariate mixed effects, comparing the exposure (SSC type on the clinic visit day) to the binary SNOT-22 survey response. The fixed-effect covariates included each patient’s visit number, age, sex, and year. A unique identification number for each patient was included as a random intercept to account for repeated measures within individuals. Odds ratios and 95% confidence intervals were derived by exponentiating the model coefficients. The resulting adjusted odds ratio (aOR) estimates the independent association between SSC category and the odds of a high SNOT-22 score while controlling for demographic, temporal, and treatment-related confounders. This analysis was repeated for the various SNOT-22 subdomains: rhinologic, extranasal, ear/facial, psychological, and sleep. For each subdomain, one standard deviation above the mean was used as the threshold to identify high SNOT-22 scores. Analyses were run in R-Studio using the “lme4” package, and model coefficients were estimated via the Laplace approximation. We used a Type I error rate of 0.05 for statistical significance and 95% confidence intervals were derived from the model’s standard errors.

## Results

After applying zip code, provider, date, and diagnosis parameters, 955 individual SNOT-22 scores from 481 distinct patients were included. Demographic and baseline clinical characteristics are summarized in Table 2. The mean SNOT-22 score in the included population is 32 (median = 31, standard deviation = 18). ANOVA results (not shown) indicated no difference in mean SNOT-22 scores as a function of day-of-week (*p* = 0.192) or month (*p* = 0.134), but differences across years were statistically significant (*p* < 0.001), primarily driven by high mean scores (> 40) in 2013 and 2020 and comparatively low values (< 30) in 2016.

**Table 2:** Patient demographics and clinical characteristics. # = frequency; SD, standard deviation; AS, acute sinusitis; CAR, chronic allergic rhinitis; CNAR, chronic nonallergic rhinitis; CRSwNP, chronic rhinosinusitis with nasal polyposis; CRSsNP, chronic rhinosinusitis without nasal polyposis; STRUCT, structural defect; NOA, none of the above

Total Patients, #	481
Total SNOT-22 Surveys, #	955
Age in Years, mean (SD)	49.6 (18.7)
Male, # (%)	233 (48.4)
Ethnicity, # (%)	
Hispanic	18 (3.7)
Non-Hispanic	454 (94.4)
Diagnosis, # (%)	
AS	172 (35.8)
CAR	240 (49.9)
CNAR	161 (33.5)
CRSwNP	82 (17.0)
CRSsNP	321 (66.7)
STRUCT	129 (26.8)
NOA	57 (11.9)

High (≥ 50) SNOT-22 days were statistically significantly associated with low mean values of numerous weather variables (Supplemental Table 2). For example, at lag 0, low temperature (1 a.m., 7 a.m, 1 p.m.), wet bulb temperature (1 a.m., 7 a.m., 1 p.m, 7 p.m.), dew point temperature (7 a.m, 7 p.m.), apparent temperature (1 a.m, 1 p.m., 7 p.m.) and humidex (1 a.m., 7 a.m., 1 p.m.) were all associated with high SNOT-22 days. Temperature and humidity results were similar for a 1-day lag (i.e., weather conditions on the day prior to the clinic visit). High SNOT-22 days were also significantly associated with high sea-level pressure (1 a.m., 7 a.m., 1 p.m.). The only significant relationships detected between high SNOT-22 days and pollutant measures were daily minimum O_3_ levels at 2- and 3-day lags (Supplemental Table 3).

**Fig. 1:**
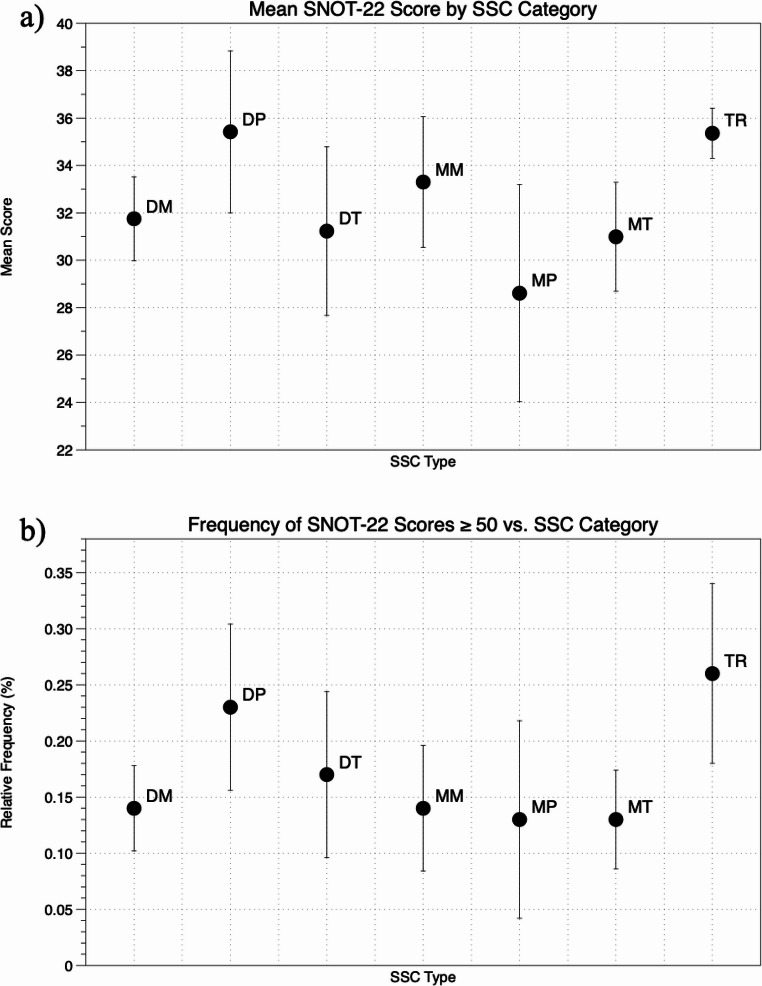
Mean SNOT-22 scores (**a**) and frequency of days with high (≥ 50) SNOT-22 scores (**b**) across spatial synoptic climatology (SSC) type. Bars represent (**a**) 2 standard errors of the mean and (**b**) 95% CIs. SSC categories: DM - dry moderate; DP - dry polar; DT - dry tropical; MM - moist moderate; MP - moist polar; MT - moist tropical; TR - transitional

The ANOVA results for SSC type indicate statistically significant differences in the frequency of high SNOT-22 scores across SSC weather types (*p* = 0.05), with high scores associated with DP and TR days and low scores linked with MP days. Figure 1 shows mean SNOT-22 scores (a) and frequency of high SNOT-22 days (b) by SSC type.

Chi-square analysis was used to compare the frequency of the seven SSC types on days with high mean SNOT-22 scores to the frequency on all other days. The results indicate different frequency distributions between the two groups (*p* = 0.01), with relatively high frequencies on DP and TR air mass days. Thus, the DP and TR air masses were combined into a single group for the purposes of calculating odds ratios (OR). 2 × 2 contingency tables compared frequencies of high and non-high SNOT-22 scores on DP and TR days vs. all other days.

For all patients taken together, odds of high SNOT-22 scores occurring on a DP or TR day vs. other weather days were significant with an OR = 2.0 [95% CI 1.4, 2.8]. The ORs weakened as a function of lag (1-day lag OR = 1.4 [0.99, 2.03]; 2-day lag OR = 0.9 [0.59, 1.29], 3-day lag OR = 1.3 [0.88, 1.83]) (Table 3).

**Table 3:** Odds ratios (95% CIs) of high SNOT-22 days (mean scores ≥ 50) on days that were either DP or TR versus other days. Significant values (CIs that exclude 1.0) are bolded. ORs were computed from the 2 × 2 contingency tables, and CIs were estimated using Wald’s method. AS, allergic sinusitis; CR, chronic rhinitis; CAR, chronic allergic rhinitis; CNAR, chronic nonallergic rhinitis; CRS, chronic rhinosinusitis; CRSwNP, chronic rhinosinusitis with nasal polyposis; CRSsNP, chronic rhinosinusitis without nasal polyposis; STRUCT, structural defect; NOA, none of the above; n/a, not applicable

Diagnosis	Same Day	1-Day Lag	2-Day Lag	3-Day Lag
All Diagnoses	**2.0 (1.4**,** 2.8)**	1.4 (1.0, 2.0)	0.9 (0.6, 1.3)	1.3 (0.9, 1.8)
AS	1.6 (0.9, 2.8)	1.5 (0.9, 2.6)	1.0 (0.6, 1.8)	**2.1 (1.2**,** 3.6)**
CR	**1.8 (1.2**,** 2.9)**	1.1 (0.7, 1.8)	0.9 (0.6, 1.5)	**1.7 (1.1**,** 2.8)**
CAR	0.7 (0.4, 1.3)	0.6 (0.4, 1.1)	1.0 (0.6, 1.7)	0.9 (0.6, 1.6)
CNAR	1.0 (0.6, 1.8)	0.8 (0.4, 1.4)	1.0 (0.5, 1.8)	0.8 (0.4, 1.5)
CRS	**1.8 (1.2**,** 2.9)**	1.1 (0.7, 1.8)	0.9 (0.6, 1.5)	**1.7 (1.1**,** 2.8)**
CRSwNP	**2.1 (1.1**,** 4.0)**	**2.2 (1.1**,** 4.2)**	**2.3 (1.2**,** 4.3)**	1.9 (0.98, 3.7)
CRSsNP	**1.8 (1.1**,** 3.0)**	1.3 (0.8, 2.1)	1.1 (0.6, 1.8)	1.6 (0.99, 2.6)
STRUCT	1.2 (0.6, 2.8)	0.8 (0.3, 2.0)	1.5 (0.6, 3.4)	1.5 (0.7, 3.2)
NOA	n/a	4.5 (0.3, 78.2)	n/a	n/a

**Fig. 2:**
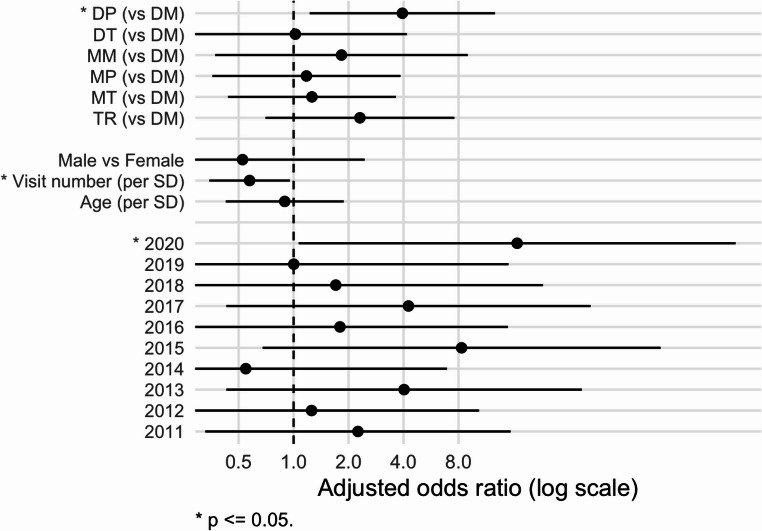
Adjusted odds ratios for SSC, demographics, visit number, and year, derived from multivariate logistic regression. Statistically significant results (alpha ≤ 0.05) are indicated by an asterisk. Confidence intervals are +/– one standard error. SSC categories: DM - dry moderate; DP - dry polar; DT - dry tropical; MM - moist moderate; MP - moist polar; MT - moist tropical; TR - transitional. SSC results are referenced to DM; age and visit number are relative to one standard deviation (SD)

The cold, dry air association was confirmed via the logistic regression results (Fig. 2). The occurrence of DP air on the day of the clinic visit was associated with 4 times higher odds of a high SNOT-22 score (aOR = 3.94 [1.23, 12.70]) compared to a reference (DM) day, while controlling for demographics and year. Results for TR days showed an elevated but non-significant risk (aOR = 2.31 [0.70, 7.60]) that is consistent with the DP effect. We also found a treatment effect whereby patients who made multiple visits showed decreased odds of having a high SNOT-22 score (aOR = 0.57 [0.35, 0.95]). Finally, in 2020, the year of the COVID-19 outbreak, odds of reporting high SNOT-22 scores were elevated. We did not find significant effects of age or gender.

Subgroup analyses by diagnosis identified statistically significant ORs for high mean SNOT-22 days in both chronic rhinitis and chronic rhinosinusitis patients at lag-0 (OR = 1.8 [1.2, 2.9] for both) and at lag-3 (OR = 1.7 [1.1, 2.8] for both). Analysis further identified significant ORs for CRSwNP and CRSsNP at lag 0, and for CRSwNP at lags 1 and 2. Finally, allergic sinusitis also showed a statistically significant effect at lag 3 (Table 3). However, given the small subgroup sample sizes, these results should be interpreted with caution. A logistic regression by subgroup did not confirm these findings (Supplemental Fig. 1).

Analysis by SNOT-22 symptom subdomain showed similar results (Table 4). ORs of high SNOT-22 days occurring with DP or TR weather were significant for the extranasal (OR = 1.5 [1.0, 2.1]); ear/facial (OR = 1.8 [1.0, 2.1]); psychological (OR = 1.6 [1.1, 2.3]); and sleep (OR = 1.8 [1.3, 2.6]) subdomains. The OR just missed significance for the rhinologic subdomain (OR = 1.3 [0.9, 1.9]); however, significance was demonstrated at lag-1 (OR = 1.6 [1.1, 2.3]).

**Table 4:** Odds ratios (95% CIs) of high (≥ threshold) mean SNOT-22 subdomain scores on either DP or TR vs. other days at 0–3-day lag. Significant values (CIs that do not include 1) are bolded. ORs were computed from the 2 × 2 contingency tables, and CIs were estimated using Wald’s method. The threshold represents 1 standard deviation above the mean SNOT-22 score for each category across all patients and diagnoses. # represents the number of SNOT-22 surveys with subdomain scores ≥ threshold. % represents percentage of total SNOT-22 surveys with subdomain scores ≥ threshold

SNOT-22 Domain	Score threshold	# (%)	Same Day	1-Day Lag	2-Day Lag	3-Day Lag
Rhinologic	11	210 (20.2)	1.3 [0.9, 1.9]	**1.6** **[1.1**,** 2.3]**	1.0 [0.7, 1.5]	1.1 [0.7, 1.5]
Extranasal	8	193 (18.0)	**1.5** **[1.0**,** 2.1]**	1.4 [0.96, 2.0]	0.9 [0.6, 1.3]	1.0 [0.7, 1.5]
Ear/Facial	11	205 (19.7)	**1.8** **[1.3**,** 2.6]**	1.4 [0.96, 2.0]	1.0 [0.7, 1.5]	1.1 [0.8, 1.7]
Psychological	20	189 (17.7)	**1.6** **[1.1**,** 2.3]**	**1.5** **[1.03**,** 2.1]**	0.9 [0.6, 1.4]	1.0 [0.7, 1.5]
Sleep	12	197 (18.2)	**1.8** **[1.3**,** 2.6]**	**1.5** **[1.1**,** 2.2]**	0.9 [0.6, 1.3]	1.1 [0.8, 1.6]

## Discussion

In the present study, we leverage both publicly accessible climate data and a robust collection of clinical data from the rhinology clinic at a tertiary care academic referral center to investigate the effect of weather on sino-nasal symptoms. Specifically, we ask if weather on or 1–3 days before the day of survey completion affects sinus symptoms as measured by mean daily SNOT-22 survey scores.

For all patients taken together, odds of a high SNOT-22 score on the day of clinic visit were almost four times higher when the air mass was DP on that day. This is consistent with preliminary analysis showing significant associations between high SNOT-22 days and low temperature, low humidity, and high pressure (all of which are DP characteristics). TR days were not statistically significant in the logistic regression but did exhibit significantly elevated SNOT-22 scores in the 2 × 2 contigency analysis. These days are characterized by changes in temperature and humidity, such as days with frontal passages, which typically feature cold and dry conditions at some time during the day at this location. Thus, the weak but suggestive result for TR days is consistent with the primary and much stronger DP result. Although ORs calculated at lags 1–3 failed to reach significance, subgroup analyses did exhibit some lag effects.

Analysis by diagnosis showed a significant effect on days with DP or TR weather for patients with chronic rhinitis (OR = 1.8), CRS (OR = 1.8), CRSwNP (OR = 2.1) and CRSsNP (OR = 1.8). The CRSwNP group showed a significant effect at 1- and 2-day lags whereas the CRSsNP showed only a same-day effect. These findings suggest that chronic inflammatory sino-nasal conditions may be affected by same-day weather, with some lag effect in certain subgroups. Furthermore, high SNOT-22 days for the structural and none-of-the-above subgroups did not show an association with DP or TR weather. This is pertinent as although not healthy controls, these groups may represent less-inflammatory comparators whose negative results strengthen the above conclusion.

SNOT-22 subdomain analysis showed odds of severe symptoms across all domains except rhinologic were statistically significantly higher on DP and TR days than other days (the rhinological results were significant at lag 1, however). These results suggest that cold and dry climate conditions exert diverse effects on symptomatology of sino-nasal disease. This is significant, as much of the literature discussing the role of weather in sinus symptoms focuses on sinus headache (Levine et al., 2014; Rudmik et al. 2015; Gryglas 2016).

The body of literature investigating the effects of environmental pollutants on upper airway health is more developed than that of weather conditions. Studies have shown exposure to various pollutants to be associated with sino-nasal symptoms (Riediker et al. 2001; Estévez-García et al. 2013; Mady et al. 2018a, b; Park et al. 2019; Velasquez et al. 2020) as well as upper airway inflammation measured in various in vitro settings (Kopp et al. 1999; Zhao et al. 2011; Cho et al. 2014; Xian et al., 2020). In contrast, the present study did not show a strong, consistent association between high SNOT-22 days and measured pollutant levels; however, this study was not optimally designed to detect such an effect. The study area has relatively low pollutant concentrations (American Lung Assoc., 2024) and few pollution monitors as compared to more urban locations, for example (Mady et al. 2018a, b; Velasquez et al. 2020). In the absence of more robust public pollutant monitoring, more sensitive methods might have accounted for distance from major pollutant sources or included wearable pollutant sensors. Current methods are suited to detect a climate effect as weather tends to be more uniformly distributed across an area of the studied size compared to primary pollutants that may be emitted from discrete foci with a variable geographic distribution.

Although studies describing the real-time effects of climate on sino-nasal symptoms are relatively scarce, a few studies have implicated weather (or surrogates) in sinus disease. Our results are consistent with those of Graudenz et al. (2006). Their group exposed subjects to cold, air-conditioned air that mimics the cold dry air of DP air masses while transient study exposures may mimic the changing weather conditions of TR days. Our results are also generally consistent with the del Rio et al. (2012) observed increased incidence of wintertime MRI sinus pathology, although study variables differ significantly. Braat et al. (2002) found an association with low minimum temperature and symptomology of nasal hyper-reactivity among a group of non-allergic non-infectious perennial rhinitis patients in the Netherlands. Similarly, controlled chamber experiments by Bernstein et al. (2012) identified non-allergic rhinitis exacerbations in association with both cold, dry air and short-term temperature changes—results that mirror our primary findings. These same conditions also have been found to impact patients with empty nose syndrome (Manji et al. 2019).

Our findings suggest avoidance of exposure to cold and dry conditions may improve sino-nasal symptoms. Indoor humidification in winter in climates where cold and dry air is common may likewise reduce exposure. Furthermore, controlling for climatic factors may allow for more standardized measurement of sino-nasal symptoms in both clinical practice and research.

There are several methodological strengths to the present study. We include a large clinical sample size with 10 years of study data. We investigate a patient-centered symptom survey that includes inherent subjectivity but allows for the study of patient experience in ways objective disease markers may not. This study is a product of interdisciplinary collaboration between faculty members representing the rhinology division of the otolaryngology department as well as the department of environmental sciences at a large academic research institution, allowing for diverse expert input throughout.

Limitations include the heterogeneity of patients included in the study as well as the disease processes themselves. Patients were stratified by ICD 9/10 diagnosis code for subgroup analysis without inclusion of radiographic or endoscopic data. Although multivariable logistic regression controlled for time, visit number, and demographic factors, certain treatment details and other potential modifying or confounding variables may have been excluded. It is likely that visit number is correlated with ongoing, or step-up treatment, but our sample includes some patients who made follow-up visits after discontinuing a treatment, and we cannot account for these cases in our analysis. We also do not account for diversity of treatment modalities (i.e. medical, surgical, environmental). We adjusted for repeated measures, but unmeasured confounders such as concurrent upper-respiratory infections or indoor exposures could contribute residual correlation. Sensitivity analyses excluding same-day multiple diagnoses produced nearly identical results (not shown). Although we believe the total number of surveys provides a sufficiently robust sample to support our overall results, it is likely that the subdomain results are underpowered and should therefore be considered with caution. Patient home address was used for inclusion and although SNOT-22 scores were measured during in-person clinic visits requiring proximity to weather monitors on day of measurement, methods do not guarantee proximity on lag days or measure individual patient microclimates. This is a common limitation of environmental studies that do not include wearable monitors; however, the included geography, covering 736 square miles, is meteorologically small, ensuring weather variability on a given day is minimal across the study region.

Other limitations are inherent to the SNOT-22 survey. Firstly, SNOT-22 items are sensitive, not specific, and may detect a host of other neurologic, head and neck, sleep, or mood disorders with potentially variable association with weather (La Mantia et al. 2023). Symptom overlap between sinus disease and migraine, for example, is well described as both implicate the trigeminal nerve (Eross et al. 2007; Gryglas 2016; Levine et al. 2014). Climate has also been proposed to play a role in migraine attacks and although the SNOT-22 is written to detect ‘symptoms and social/emotional consequences of your nasal disorder,’ patients may have trouble differentiating overlapping symptoms from distinct disease states (Tepper 2004; Gryglas 2016). Furthermore, the survey instructs the patient to ‘rate your problems, as they have been over the past two weeks.’ This allows symptoms prior to measured weather exposures to potentially enter our analysis which may limit internal validity. However, the strongest detected effect being present on day of SNOT-22 completion that tends to weaken as a function of lag supports our conclusions. Finally, this study was based on a long time series of data available from a single site with a modest local population. Examination of comparable data from locations with different climates and more dense populations would be useful to test generalizability of these findings.

Future work could include patient reported symptom measures specifically designed to optimally capture effects of interest. Using customized measures may limit external validity, however, and the SNOT-22’s prior validation and widespread use rendered it a useful tool for this retrospective study. Directions for future work include a comparable examination in different climates or geographies including heavily populated urban centers with higher pollution levels, incorporating wearable technology to more reliably capture experienced weather and control for experienced pollutant levels, and further elucidating mechanisms behind the effects of climate on sino-nasal symptomatology.

## Conclusions

Climate conditions are often subjectively implicated in sino-nasal symptoms despite little objective data. We show that on days with cold and dry air patients have much higher odds of reporting worse sino-nasal symptoms. This provides evidence to a popularly held belief, suggesting that weather should be considered when managing and measuring sino-nasal symptoms, and specifically that avoiding cold and dry air might improve symptom control. Future research should be undertaken in more highly polluted areas, specifically measuring individual microclimate conditions, and controlling for disease course.

## Supplementary Information

Below is the link to the electronic supplementary material.

Supplementary Material 1 (DOCX 448 KB)

## Data Availability

Access to the data used in this study is governed by the Virginia Department of Health (VDH)’s and the University of Virginia (UVA)’s Institutional Review Board (IRB) Data Management and Security Plan, which is available from the authors upon request. The self-reported health outcomes data used in this research includes confidential protected health information and is therefore not publically available, per HIPAA laws of the United States of America and the VDH and UVA IRB policies on data security.
