# Supplementary material for: Climate and sino-nasal symptoms
Source: Int J Biometeorol. 2026 May 19;70(6):165. doi: 10.1007/s00484-026-03154-z (PMC13186851; doi:10.1007/s00484-026-03154-z)

**Supplemental Tables**

Supplemental Table 1. The SNOT-22 survey instrument (Kennedy et al., 2013).


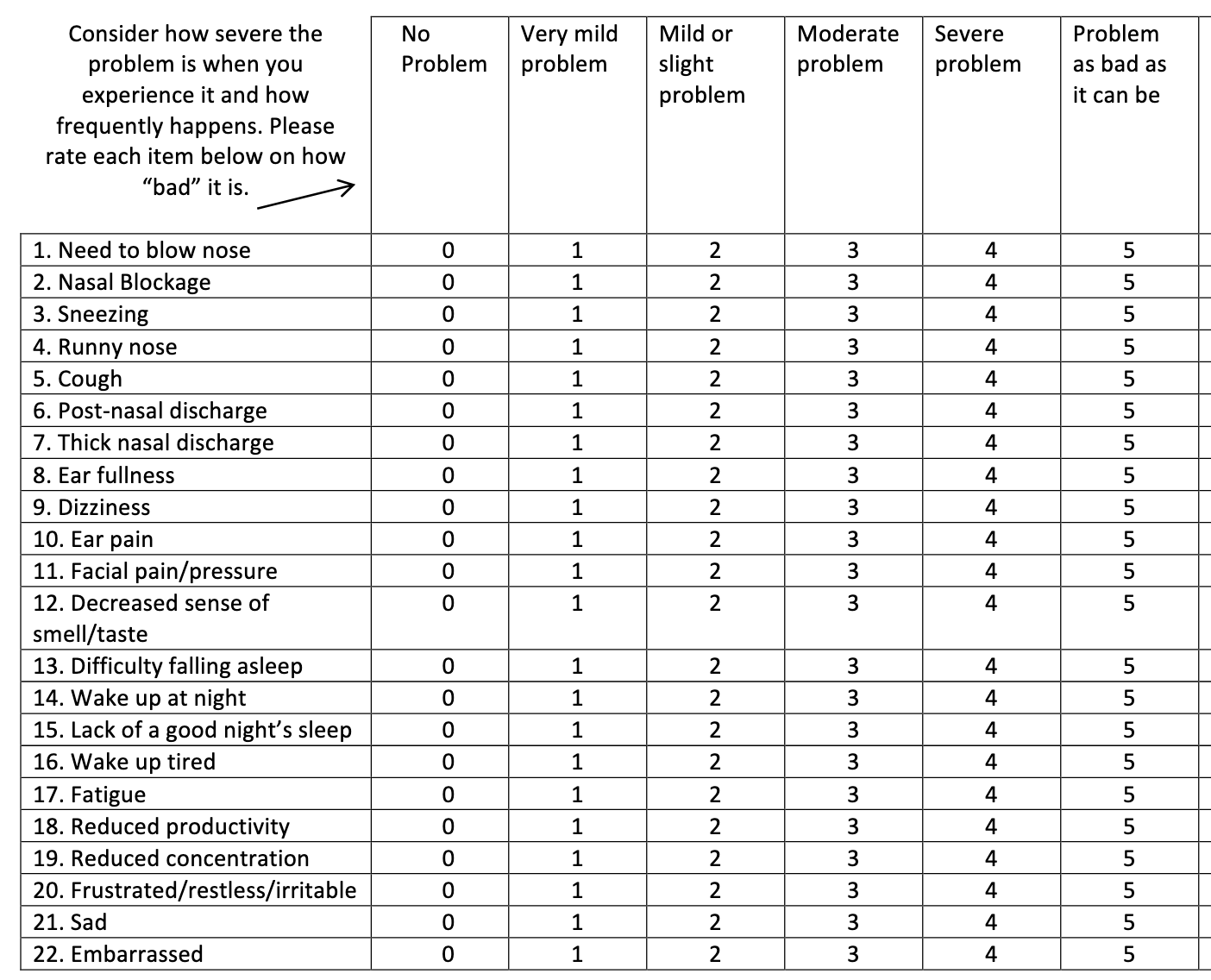


Supplemental Table 2. Summary of 2-tailed t-test results comparing individual weather variables on days with high mean SNOT-22 scores to all other days. An upward pointing arrow indicates that weather parameter had high mean values on days with high SNOT-22 scores whereas a downward pointing arrow indicates low readings.

|  | **Lag 0** |  |  |  |  | **Lag 1** |  |  |  |
| --- | --- | --- | --- | --- | --- | --- | --- | --- | --- |
|  | *1 a.m.* | *7 a.m.* | *1 p.m.* | *7 p.m.* |  | *1 a.m.* | *7 a.m.* | *1 p.m.* | *7 p.m.* |
| *Temperature* | 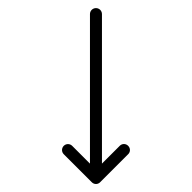 | 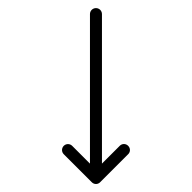 | 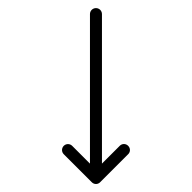 |  |  | 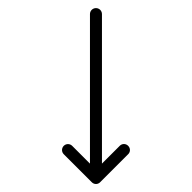 | 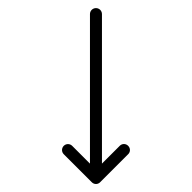 | 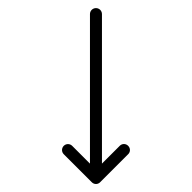 | 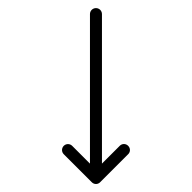 |
| *Wet Bulb* | 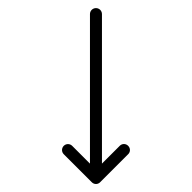 | 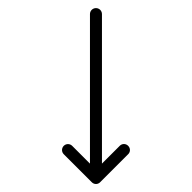 | 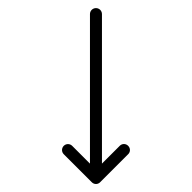 | 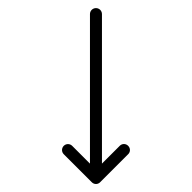 |  |  | 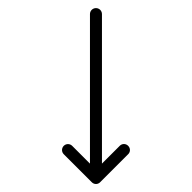 | 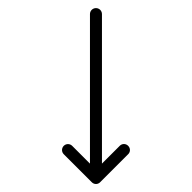 | 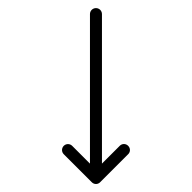 |
| *Dew Point* |  | 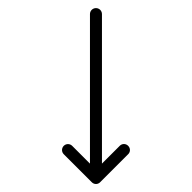 |  | 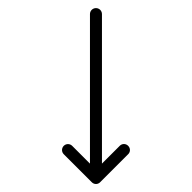 |  |  | 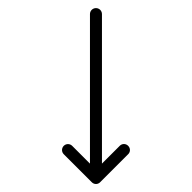 | 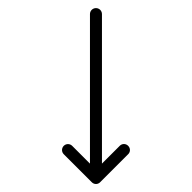 |  |
| *Pressure* |  |  |  |  |  | 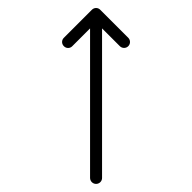 | 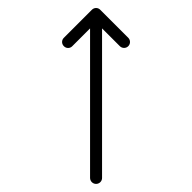 | 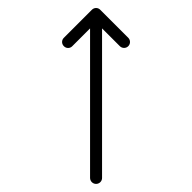 |  |
| *Wind Speed* |  |  |  | 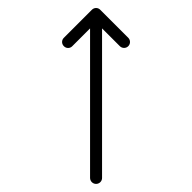 |  |  |  |  |  |
| *Apparent*  *Temperature* | 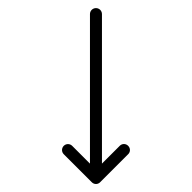 |  | 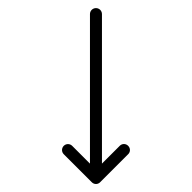 | 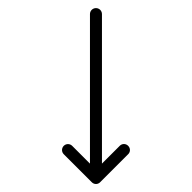 |  | 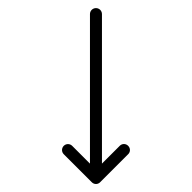 | 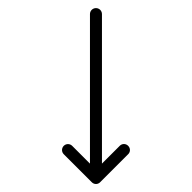 | 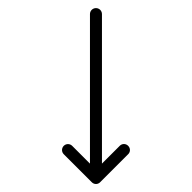 | 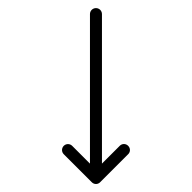 |
| *Humidex* | 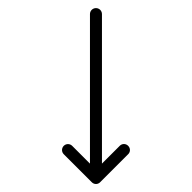 | 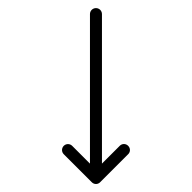 | 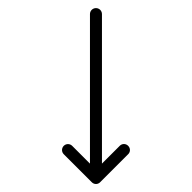 |  |  | 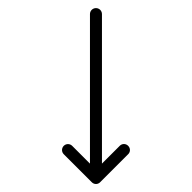 | 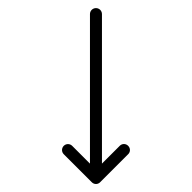 | 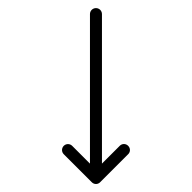 | 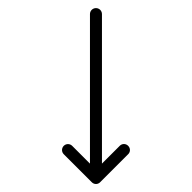 |
| *Wind Chill* |  | 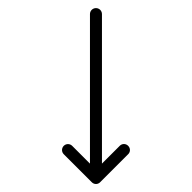 |  |  |  |  | 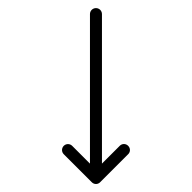 | 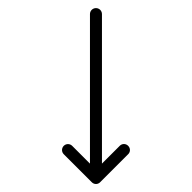 | 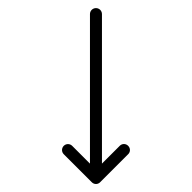 |

Supplemental Table 3. One-tailed t-test values (95% CIs) on days with high SNOT-22 scores (mean≥50) for various daily measures of ozone and particulate matter concentration. Pollutant levels are measured hourly, and 24-hour values include all daily measurements. "Day" values include only daytime observations (measured between 7 a.m. and 6 p.m. Eastern Standard Time). Significant associations are bolded.

| **Pollutant** | **Measure** | **Same Day** | **1-Day Lag** | **2-Day Lag** | **3-Day Lag** |
| --- | --- | --- | --- | --- | --- |
| Ozone (O_3_) | 24 hr Min | 1.2 (-0.6, 2.6) | 1.1 (-0.7, 2.5) | **2.3 (0.3, 3.8)** | **2.5 (0.4, 3.9)** |
|  | 24 hr Max | -0.4 (-2.3, 1.5) | -0.1 (-2.0, 1.8) | -0.4 (-2.2, 1.5) | -0.3 (-2.1, 1.6) |
|  | 24 hr Mean | 0.8 (-1.0, 2.4) | 0.4 (-1.3, 2.0) | 1.2 (-0.6, 2.5) | 1.5 (-0.4, 2.6) |
|  | Day Min | -0.05 (-2.1, 2.0) | 0.4 (-1.5, 2.3) | 0.3 (-1.6, 2.1) | 0.3 (-1.6, 2.2) |
|  | Day Max | -0.3 (-2.3, 1.7) | -0.2 (-2.2, 1.8) | -0.3 (-2.3, 1.6) | -0.2 (-2.1, 1.8) |
|  | Day Mean | 0.1 (-1.9, 2.2) | -0.4 (-1.9, 1.8) | -0.5 (-1.9, 1.8) | 0.01 (-1.8, 1.8) |
| Particulate Matter ≤ 2.5 microns (PM_2.5_) | 24 hr Min | -2.7 (-1.3, -0.2) | -2.4 (-1.2, -0.1) | -2.8 (-1.3, -0.2) | -2.8 (-1.3, -0.2) |
|  | 24 hr Max | -1.1 (-1.5, 0.4) | -1.1 (-1.6, 0.5) | -0.7 (-1.4, 0.7) | -1.3 (-1.6, 0.4) |
|  | 24 hr Mean | -2.2 (-1.2, -0.1) | -1.6 (-1.0, 0.1) | -1.7 (-1.1, 0.1) | -2.2 (-1.2, -0.1) |
|  | Day Min | -3.0 (-1.4, -0.3) | -2.1 (-1.2, -0.1) | -2.4 (-1.4, -0.2) | -2.8 (-1.5, -0.3) |
|  | Day Max | -1.1 (0.0, 0.4) | -0.8 (-1.3, 0.6) | -0.7 (-1.4, 0.6) | -1.8 (-1.8, 0.1) |
|  | Day Mean | -2.4 (-1.4, -0.2) | -1.1 (-1.0, 0.3) | -1.7 (-1.1, 0.1) | -2.5 (-1.5, -0.2) |

Supplemental Figure 1. Adjusted odds ratios from multivariate mixed-effects logistic regression by diagnosis. Confidence intervals are +/– one standard error. SSC categories: DM - dry moderate; DP - dry polar; DT - dry tropical; MM - moist moderate; MP - moist polar; MT - moist tropical; TR - transitional. SSC weather types are referenced to DM.


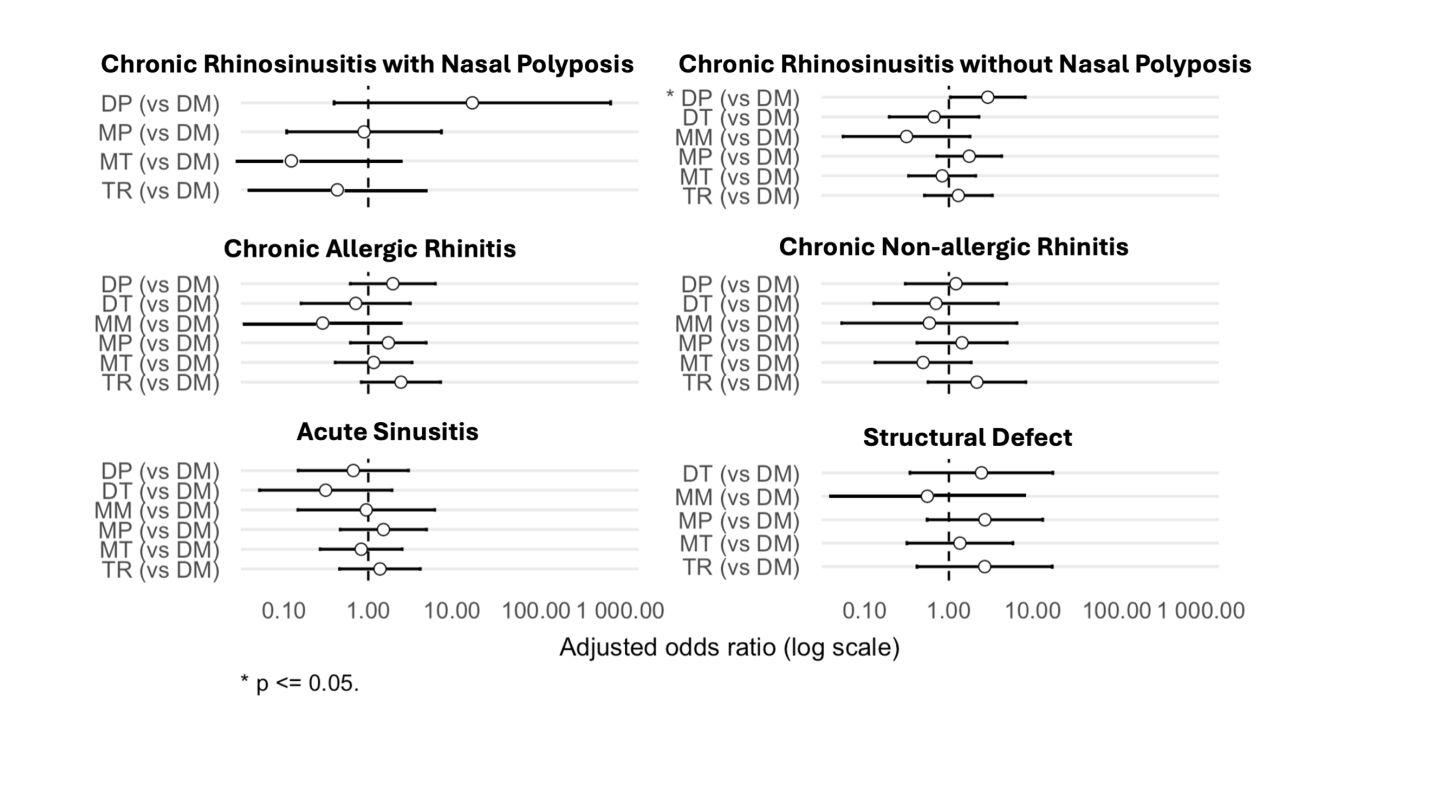

Supplement: Supplementary file 1 — Supplementary Material 1 (DOCX 448 KB) [file 484_2026_3154_MOESM1_ESM.docx]
